# Supplementary material for: Theory, practice, and conservation in the age of genomics: The Galápagos giant tortoise as a case study
Source: Evol Appl. 2017 Oct 23;11(7):1084–93. doi: 10.1111/eva.12551 (PMC6050186; doi:10.1111/eva.12551)
Supplement: Supplementary file 1 [file EVA-11-1084-s001.docx]

**Supplementary Material**

1. Null distribution of F_ST_ estimates

Our analyses of the ddRAD-seq SNP data revealed high pairwise F_ST_ values between all three population pairs (see main text; Table 2). To complement our bootstrapping method of testing significance, we created artificial groupings of individuals from different species, with the null hypothesis that if the *Chelonoidis* from the Galápagos islands were panmictic F_ST_ estimates for these artificial groupings should not be significantly different from F_ST_ estimates for our *a priori* assignment of individuals to their respective species designations. To minimize the effect of sample size but maintain equal contributions from each species, we created 2000 artificial groups of nine individuals, with three drawn randomly from each species. The average F_ST_ for these randomly drawn “populations” was -0.018, with 95% of the calculated F_ST_ values falling between -0.024 and -0.009 (Supplementary Figure S1). This indicates that the F_ST_ values calculated between the three species are highly significant, given that F_ST_ analyses of randomly-generated artificial populations of the same individuals give near-zero values.


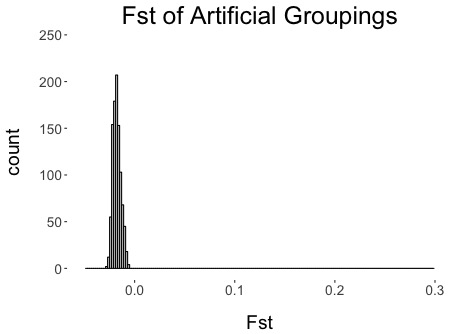


Figure S1. Histogram of F_ST_ values from 1000 pairwise comparisons of artificial populations generated as a mixture of individuals from the three species of Galápagos giant tortoises under study, *C. becki*, *C. porteri*, and *C. vandenburghi*. Near-zero F_ST_ values show that this method for estimating F_ST_ values is robust to false positives that would arise from random combinations of individuals with no population structure.

1. ddRAD-seq subsampling for small sample size

**Table S1 A—C.** Summary of pairwise *F*_ST_ estimates for 1000 subsamples of each sample size (n=2, 3, and 5). Estimates were ordered from lowest to highest, and the 1^st^ (lowest), 25^th^ (2.5^th^ percentile), 250^th^ (25^th^ percentile), 500^th^ (50^th^ percentile), 750^th^ (75^th^ percentile), 975^th^ (97.5^th^ percentile), and 1000^th^ (highest) estimates were reported for each sample size. The *F*_ST_ value calculated using all ten samples from each population is noted at the top of each table. PBL, CRU, and VA refer to the *C. becki*, *C. porteri*, and *C. vandenburghi* populations used in this study.

A) PBL vs. CRU (10 sample *F_ST_ = 0.169*)

|  | **N = 2** | **N = 3** | **N =5** |
| --- | --- | --- | --- |
| **1** | 0.130 | 0.143 | 0.154 |
| **25 (2.5%)** | 0.139 | 0.149 | 0.157 |
| **250 (25%)** | 0.152 | 0.158 | 0.165 |
| **500 (50%)** | 0.160 | 0.166 | 0.169 |
| **750 (75%)** | 0.179 | 0.181 | 0.174 |
| **975 (97.5%)** | 0.289 | 0.204 | 0.184 |
| **1000** | 0.327 | 0.217 | 0.190 |
| **Mean** | 0.170 | 0.170 | 0.170 |

B) PBL vs. VA (10 sample *F_ST_ = 0.181)*

|  | **N = 2** | **N = 3** | **N =5** |
| --- | --- | --- | --- |
| **1** | 0.134 | 0.147 | 0.158 |
| **25 (2.5%)** | 0.142 | 0.152 | 0.162 |
| **250 (25%)** | 0.156 | 0.164 | 0.171 |
| **500 (50%)** | 0.171 | 0.173 | 0.178 |
| **750 (75%)** | 0.184 | 0.187 | 0.185 |
| **975 (97.5%)** | 0.337 | 0.227 | 0.195 |
| **1000** | 0.371 | 0.236 | 0.204 |
| **Mean** | 0.178 | 0.177 | 0.178 |

C) CRU vs. VA (10 sample *F_ST_ = 0.233*)

|  | **N = 2** | **N = 3** | **N =5** |
| --- | --- | --- | --- |
| **1** | 0.208 | 0.215 | 0.222 |
| **25 (2.5%)** | 0.212 | 0.220 | 0.225 |
| **250 (25%)** | 0.224 | 0.227 | 0.229 |
| **500 (50%)** | 0.230 | 0.232 | 0.232 |
| **750 (75%)** | 0.239 | 0.237 | 0.235 |
| **975 (97.5%)** | 0.265 | 0.253 | 0.241 |
| **1000** | 0.285 | 0.261 | 0.244 |
| **Mean** | 0.232 | 0.233 | 0.232 |

Figure S2. Histograms of pairwise F_ST_ estimates from 1000 subsamples of populations of each sample size (n=2, 3, and 5). The red line in each panel indicates the F_ST_ estimate using 10 individuals from each species. PBL, CRU, and VA refer to the population *of C. becki*, *C. porteri*, and *C. vandenburghi* used in this study.

A)


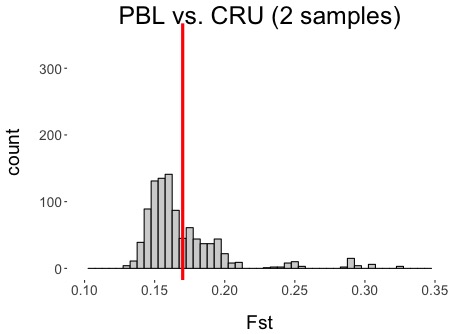

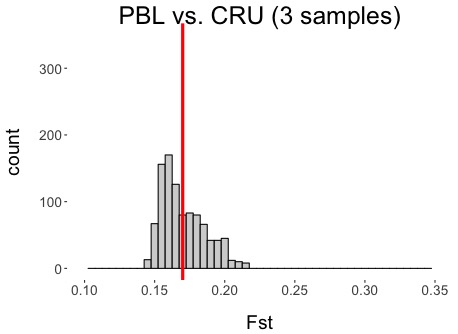


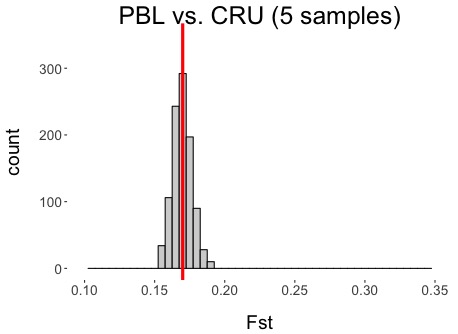


B)


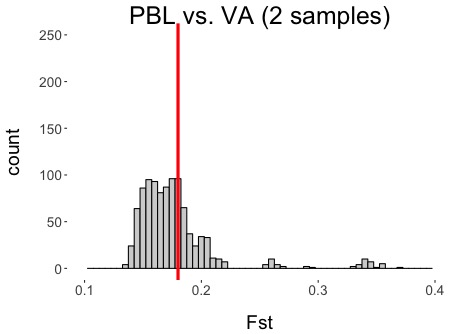

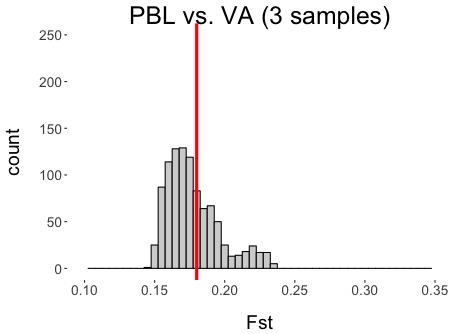


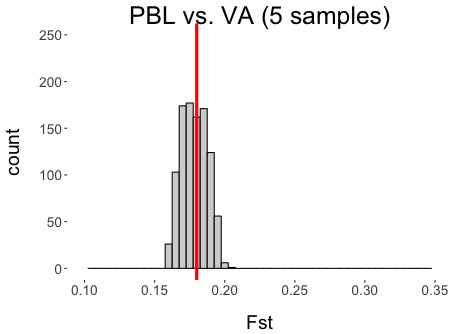


C)


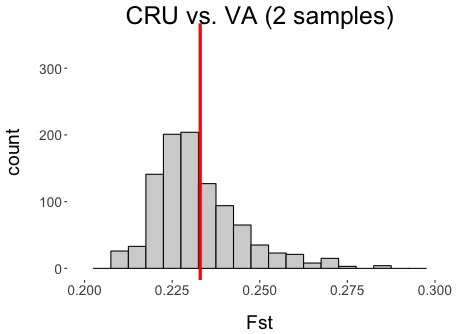

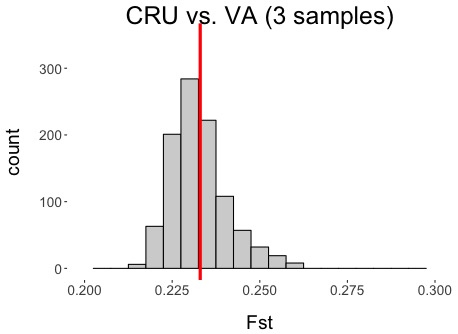


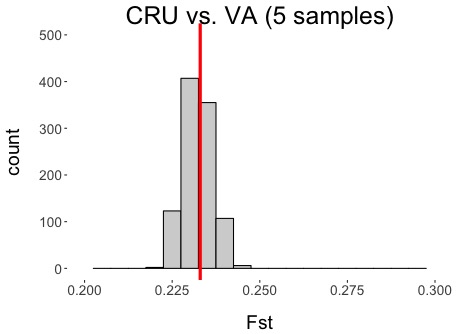


1. ddRAD-seq subsampling for different number of loci

To test for the statistical power of our SNPs to detect population structure, we subsampled our ddRAD-seq SNP data set, selecting random sets of 25, 50, 100, 200, 500, 1000, 5000, and 10,000 SNPs. Pairwise *F*_ST_ estimates were then calculated using all samples (n=10 per population). This process was repeated 1000 times for each number of SNPs to create a distribution of estimates.

Table S2 A – C. Summary of pairwise *F*_ST_ estimates for 1000 subsamples of each SNP set size (25, 50, 100, 200, 500, 1000, 5000, 10,000 SNPs). Estimates were ordered from lowest to highest, and the 1^st^ (lowest), 25^th^ (2.5^th^ percentile), 250^th^ (25^th^ percentile), 500^th^ (50^th^ percentile), 750^th^ (75^th^ percentile), 975^th^ (97.5^th^ percentile), and 1000^th^ (highest) estimates were reported for each sample size. The *F*_ST_ value calculated using all 23,057 SNPs is noted at the top of each table. PBL, CRU, and VA refer to the *C. becki*, *C. porteri*, and *C. vandenburghi* populations used in this study. Number of SNPs is listed along the top of each column.

A) PBL vs. CRU (Full set *F_ST_ = 0.169*)

|  | **25** | **50** | **100** | **200** | **500** | **1000** | **5000** | **10,000** |
| --- | --- | --- | --- | --- | --- | --- | --- | --- |
| **1** | 0.008 | 0.046 | 0.070 | 0.091 | 0.119 | 0.131 | 0.154 | 0.159 |
| **25 (2.5%)** | 0.054 | 0.082 | 0.100 | 0.116 | 0.136 | 0.146 | 0.159 | 0.162 |
| **250 (25%)** | 0.107 | 0.124 | 0.144 | 0.149 | 0.156 | 0.160 | 0.166 | 0.167 |
| **500 (50%)** | 0.150 | 0.158 | 0.166 | 0.167 | 0.168 | 0.168 | 0.169 | 0.169 |
| **750 (75%)** | 0.205 | 0.195 | 0.194 | 0.186 | 0.179 | 0.177 | 0.173 | 0.172 |
| **975 (97.5%)** | 0.344 | 0.283 | 0.255 | 0.224 | 0.206 | 0.194 | 0.180 | 0.176 |
| **1000** | 0.443 | 0.338 | 0.328 | 0.275 | 0.232 | 0.214 | 0.187 | 0.181 |
| **Mean** | 0.163 | 0.165 | 0.170 | 0.168 | 0.168 | 0.169 | 0.169 | 0.169 |

B) PBL vs. VA (10 sample *F_ST_ = 0.181)*

|  | **25** | **50** | **100** | **200** | **500** | **1000** | **5000** | **10,000** |
| --- | --- | --- | --- | --- | --- | --- | --- | --- |
| **1** | 0.013 | 0.038 | 0.068 | 0.102 | 0.117 | 0.138 | 0.164 | 0.167 |
| **25 (2.5%)** | 0.049 | 0.078 | 0.102 | 0.126 | 0.145 | 0.152 | 0.169 | 0.174 |
| **250 (25%)** | 0.108 | 0.134 | 0.149 | 0.161 | 0.168 | 0.172 | 0.177 | 0.178 |
| **500 (50%)** | 0.160 | 0.170 | 0.178 | 0.180 | 0.180 | 0.181 | 0.181 | 0.181 |
| **750 (75%)** | 0.215 | 0.214 | 0.205 | 0.202 | 0.192 | 0.190 | 0.184 | 0.183 |
| **975 (97.5%)** | 0.336 | 0.305 | 0.265 | 0.243 | 0.217 | 0.207 | 0.191 | 0.188 |
| **1000** | 0.481 | 0.355 | 0.341 | 0.282 | 0.245 | 0.230 | 0.200 | 0.194 |
| **Mean** | 0.169 | 0.176 | 0.179 | 0.182 | 0.180 | 0.181 | 0.181 | 0.181 |

C) CRU vs. VA (10 sample *F_ST_ = 0.233*)

|  | **25** | **50** | **100** | **200** | **500** | **1000** | **5000** | **10,000** |
| --- | --- | --- | --- | --- | --- | --- | --- | --- |
| **1** | -0.002 | 0.042 | 0.083 | 0.121 | 0.145 | 0.177 | 0.206 | 0.212 |
| **25 (2.5%)** | 0.051 | 0.091 | 0.133 | 0.152 | 0.183 | 0.196 | 0.218 | 0.223 |
| **250 (25%)** | 0.134 | 0.168 | 0.192 | 0.205 | 0.213 | 0.220 | 0.228 | 0.229 |
| **500 (50%)** | 0.196 | 0.217 | 0.230 | 0.229 | 0.230 | 0.232 | 0.233 | 0.233 |
| **750 (75%)** | 0.274 | 0.273 | 0.265 | 0.258 | 0.247 | 0.245 | 0.238 | 0.236 |
| **975 (97.5%)** | 0.438 | 0.389 | 0.338 | 0.312 | 0.280 | 0.265 | 0.248 | 0.242 |
| **1000** | 0.591 | 0.515 | 0.427 | 0.340 | 0.308 | 0.283 | 0.252 | 0.248 |
| **Mean** | 0.211 | 0.223 | 0.230 | 0.230 | 0.231 | 0.232 | 0.233 | 0.233 |

Figure S3 A – C. Boxplots of pairwise F_ST_ calculations using different numbers of randomly drawn SNPs (25, 50, 100, 200, 500, 1000, 5000, 10,000) and all 10 individuals per population. Horizontal black line on the plot shows F_ST_ calculated from all individuals and all SNPs in the data set. Lower hinge corresponds to first quartile (25^th^ percentile); upper hinge corresponds to third quartile (75^th^ percentile). Whiskers indicate points within 1.5 times the interquartile range (IQR), with outliers indicated as points beyond that range.

A)

B)

C)

1. Loire et al. (2013) subsampling scheme

In Loire et al. (2013), the authors analyze transcriptome-derived SNPs in five Galápagos giant tortoise samples from three named species—three individuals belonging to PBL (*C. becki*), one belonging to CRU (*C. porteri*), and one belonging to VA (*C. vandenburghi*)—but fail to find significant population structure. This is in stark contrast to prior genetic studies using mitochondrial DNA and microsatellites, and to the current study using ddRAD SNPs, which all found significant structure among these samples from the three species. The discrepancy between these studies and Loire et al. (2013) was most likely due to their design in which they combined samples of tortoises from two distinct species (*C. porteri* (CRU) and *C. vandenburghi* (VA)) and treated them as belonging to one population from one species. Their reasoning for this grouping was that CRU and VA belong to a different mtDNA phylogenetic clade than PBL (see supplementary materials section VII; e.g. Figure S6).

We subsampled our own dataset to mimic the Loire et al. (2013) design. We generated 1000 artificial populations that each consisted of a single sample from the *C. vandenburghi* population (VA) and a single sample from the *C. porteri* population (CRU). We then ran pairwise F_ST_ tests comparing these artificial populations to 1000 populations consisting of three randomly drawn samples from the *C. becki* population (PBL). We conducted the comparisons using our set of SNPs common to all individuals (23,057 SNPs) and a randomly drawn set of 1000 of those SNPs, which more closely mirrors the Loire et al. (2013) study design, as it was based on 769 to 1,041 SNPs.

A)


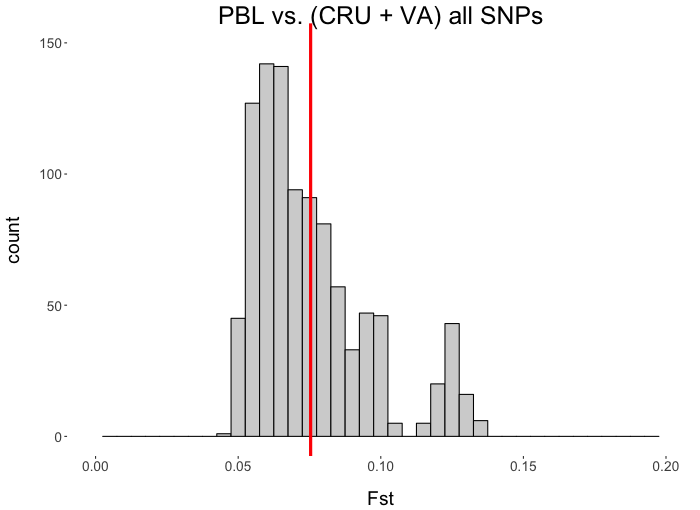


B)


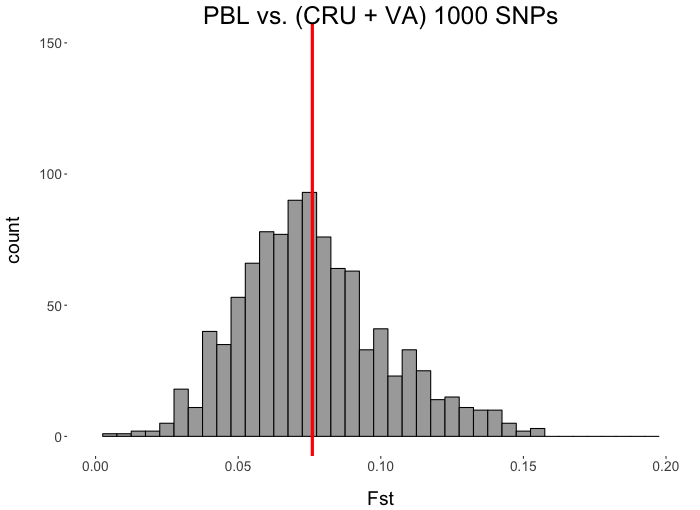


Figure S4. Histograms of 1000 F_ST_ estimates from subsamples mimicking the grouping of Loire et al. (2013). (A) Distribution when 23,057 SNPs are used in the F_ST_ estimate (95%: 0.052 – 0.127, mean: 0.075). (B) Distribution when 1000 randomly drawn SNPs are used in the F_ST_ estimate (95%: 0.031 – 0.134, mean: 0.076). Red line indicates the mean F_ST_ estimate. PBL, CRU, and VA refer to the population *of C. becki*, *C. porter*i, and *C. vandenburghi* used in this study.

1. SNP number and inclusion

Table S3. Pairwise F_ST_ values between given population pairs. Above the diagonal, values calculated using all loci common to the population pair (See Table 1 in the Main Text). Below the diagonal, values calculated using loci with no missing data common to all three populations (n = 23,057).

|  | PBL  (*C. becki*) | CRU  (*C. porteri*) | VA  (*C. vandenburghi*) |
| --- | --- | --- | --- |
| PBL  (*C. becki*) | xx | 0.171 | 0.186 |
| CRU  (*C. porteri*) | 0.169 | xx | 0.244 |
| VA  (*C. vandenburghi*) | 0.181 | 0.233 | xx |

1. Structure results

A)

B) C)

Figure S5: Structure plot summarizing the genetic assignments of the 30 Galápagos giant tortoises used in this study based on the 23,057 SNPs common to all the individuals (A). Each individual is represented as a vertical bar, with colors denoting different genetic clusters. The proportion of color in a bar is equal to the ancestry to that cluster. The optimal number of genetic clusters (K) was chosen using both the mean log likelihood values (B) and the ΔK statistic (C). Optimal K is indicated by the peak in each graph (B and C), for both methods K = 3. Error bars in plot B represent standard deviations. PBL, CRU, and VA refer to the populations of *C. becki*, *C. porter*i, and *C. vandenburghi* used in this study

1. Reanalysis of Loire et al. (2013) RNA-sequencing data

Through NCBI’s Sequence Read Archive, we downloaded RNA sequencing data from the five Galápagos giant tortoise samples generated by Loire et al. (2013), accession numbers SRS509366 - SRS509370. We used the adapter and quality trimming wrapper Trim Galore! (<http://www.bioinformatics.babraham.ac.uk/projects/trim_galore/>), and aligned the sequencing data to a draft genome of *Chelonoidis abingdonii* using TopHat v. 2.1.1 (Kim et al. 2013), allowing for a maximum of two mismatches from the reference sequence. We then used a combination of samtools and bcftools (Li 2011) to call SNPs in all five samples against the reference genome. In vcftools (Danecek et al. 2011), we filtered SNPs by the following criteria: 1) present in all samples (i.e. no missing data), 2) minimum QUAL (phred-scaled quality score) of 50, 3) minor allele frequency of 0.05. This produced a final dataset of 18,462 SNPs.

We calculated the F_ST_ for the groupings used by Loire et al. (2013) within vcftools. This gave an estimate of 0.054. We then ran the same F_ST_ estimate on the other nine possible combinations of samples in a 3:2 separation. These permutation estimates ranged from -0.024 to 0.017.

We performed PCA on the data using the indpca function in the hierfstat package version 0.04-22 in R (Goudet 2005). We plotted the Principal Component 1 against Principal Component 2 for the five samples. The PCA clearly clusters the three PBL (*C. becki*) samples together, while separating the CRU (*C. porteri*) and VA (*C. vandenburghi*) samples from each other and from the PBL ones (Figure S6). Most notably, the pattern of sample differentiation in this PCA analysis is qualitatively the same as the PCA we performed on our ddRAD-seq dataset using 10 samples from each of the three populations (Figure 3, main text).

Figure S6. Plot of Principal Components 1 and 2 from a Principal Component Analysis (PCA) of SNP data from the five Galápagos giant tortoise RNA-seq data generated by Loire et al. (2013). PBL (n=3), CRU (n=1, and VA (n=1) refer to the population *of C. becki*, *C. porter*i, and *C. vandenburghi* and the numbers after the symbol identify different individuals in the study.


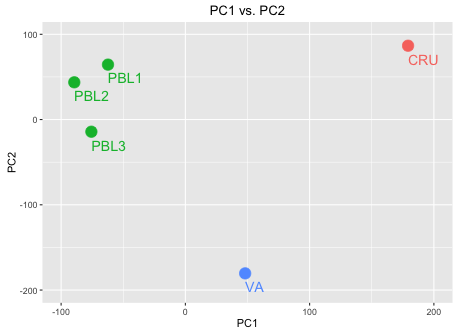


1. Supporting microsatellite and mitochondrial DNA analyses

More than a decade and a half of research has established patterns of genetic differentiation and divergence among groups of Galápagos giant tortoises. This includes screening for variation at several mitochondrial DNA (mtDNA) regions (Caccone et al. 2002; Beheregaray et al. 2004) and microsatellite loci (Ciofi et al. 2002; Beheregaray et al. 2003a; Ciofi et al. 2006), for both extant and extinct taxa though use of ancient DNA (Russello et al. 2007; Poulakakis et al. 2008). Analyses of these data have included the application of statistical models to infer dispersal and demography (Beheregaray et al. 2003b; Beheregaray et al. 2004; Poulakakis et al. 2012; Edwards et al. 2014; Garrick et al. 2015), and outcomes of analyses have been used to help guide conservation and management (Beheregaray et al. 2003b; Burns et al. 2003; Milinkovitch et al. 2004; Milinkovitch et al. 2007; Russello et al. 2007; Benavides et al. 2012; Edwards et al. 2013; Poulakakis et al. 2015).

Using population level sampling, these lines of evidence have shown that the radiation of Galápagos giant tortoises is consistent with the geologic history of the Islands, generally following the "progression rule" scenario (i.e., step-wise colonization from oldest to youngest islands (Beheregaray et al. 2003a; Parent et al. 2008; Poulakakis et al. 2012). Though there has been human-mediated translocations of individuals among islands over the past several decades (Caccone et al. 2002; Russello et al. 2007; Garrick et al. 2012; Poulakakis et al. 2012; Edwards et al. 2013), the history of the radiation is dominated by rare inter-island dispersal and intra-island vicariant events (e.g. volcanic eruptions, movement of island (Beheregaray et al. 2003b; Beheregaray et al. 2004; Poulakakis et al. 2012)).

These results are summarized in Figure S7. A phylogenetic tree based on mtDNA control region sequences based on 123 haplotypes from all populations of the extant and extinct species highlights that there is substantial divergence among haplogroups, which largely correspond to the different islands in the archipelago (Figure S7A). Of particular note is the reciprocal monophyly of the two clades containing the lineages of tortoises from *C. vandenburghi* from Volcano Alcedo in the central parts of Isabela Island (VA, Fig. 1) and from *C. porteri*, from the La Caseta population on Santa Cruz Island (CRU, Fig. 1). These lineages are also differentiated with respect to their nuclear genomes, as indicated by the STRUCTURE diagram (Fig. S7B). Thus, grouping them together as in the Loire et al. (2013) for the purpose of population genetic analyses would be expected to generate substantial within-group Wahlund effect, and depress estimates of among-group divergence.

To illustrate this we re-examined F_ST_ estimates from previously collected mtDNA sequences and microsatellite markers from all extant populations of Galápagos giant tortoises, using the data from Garrick et al. (2015). Details of the taxa and number of samples for each marker are presented in Table S4. The mtDNA sequence data corresponds to 704bp of the mtDNA control region, while the microsatellite data represents 12 dinucleotide loci (Russello et al. 2007; Poulakakis et al. 2008; Benavides et al. 2012; Garrick et al. 2012; Poulakakis et al. 2012; Edwards et al. 2013; Garrick et al. 2014). Note that these samples include all of the individuals from VA and CRU, as well as 6 of the 10 from PBL used in the ddRAD dataset (see Main Text). This dataset of 266 mtDNA sequences and multi-locus genotypes from 273 individuals had been pruned of 21 and 28 individuals respectively that either showed (1) evidence of strong admixture in their microsatellite-based ancestry assignment, (2) discordance between their sampling location and the island of origin of their mitochondrial haplotype. These individuals are likely recent migrants or the progeny of human-mediated translocations and therefore not appropriate for assessing divergences among lineages (Garrick et al. 2014). However, to evaluate if their inclusion would reduce the F_ST_ estimates, here we repeated the analyses including the omitted individuals for which both mtDNA and microsatellite data were available (Table S4).

For both classes of genetic data types, F_ST_ was calculated for two datasets. In the first we grouped individuals based on their geographic sampling location, which also reflect their species status. In the second we mimicked the grouping of Loire et al. (2013) binning together the samples from Volcano Alcedo (VA, *C. vandenburghi*) and La Caseta (CRU, *C. porteri*) populations, as if they belonged to a single panmictic population. F_ST_ among mtDNA sequences was calculated with Arlequin version 3.5.5 (Excoffier et al. 2005). In this case we used conventional F-statistics from haplotype frequencies. For the microsatellite loci we calculated pairwise F_ST_ using GenePop version 4.2 (Raymond and Rousset 1995; Rousset 2008), as implemented by GenePop on the Web (http://genepop.curtin.edu.au/). All settings were left at default values.

Using grouping based on sampling location all of the populations were highly differentiated at both marker types (Table S5). In particular, F_ST_ values between the two population samples from *C. porteri* and *C. vandenburghi* (CRU and VA) are 0.439 and 0.202 for mtDNA and microsatellites, respectively. Similarly, both populations are differentiated from the population sample from *C. becki*, PBL, with F_ST_ values of 0.721 and 0.163 for VA, and 0.466 and 0.137 for CRU at mtDNA and microsatellites, respectively. Pooling together the samples of the two species (VA and CRU) depresses the F_ST_ values relative to PBL, lowering them to 0.446 and 0.094 (Table S6). Including individuals with admixed genomes caused point estimates involving those taxa to decrease. When the two samples from VA and CRU are kept separate the F_ST_ values decrease on average by 0.191 and 0.026 for mtDNA and microsatellites, respectively (Table S7); when they are pooled together the F_ST_ values decrease on average by 0.193 and 0.025 for mtDNA and microsatellites, respectively (Table S8).

**Table S4:** Taxonomic and population sampling of Galápagos giant tortoises used for calculation of F_ST_ with mtDNA control region sequences and microsatellite genotypes.

|  |  |  |  | Excluding Admixed | | Including Admixed | |
| --- | --- | --- | --- | --- | --- | --- | --- |
| Species Name | Population | Symbol | Island | N. mtDNA | N. microsat | N. mtDNA | N. microsat |
|  |  |  |  |  |  |  |  |
| *C. hoodensis* | Española | ESP | Española | 15 | 15 | 15 | 15 |
| *C*. *chathamensis* | San Cristóbal | SCR | San Cristóbal | 19 | 19 | 19 | 19 |
| *C*. *donfaustoi* | Cerro Fatal | CF | Santa Cruz | 20 | 21 | 20 | 21 |
| *C. porteri* | La Caseta | CRU | Santa Cruz | 23 | 25 | 23 | 25 |
| *C. ephippium* | Pinzón | PZ | Pinzón | 27 | 24 | 27 | 24 |
| *C. darwini* | Santiago | AGO | Santiago | 21 | 22 | 23 | 24 |
| *C. microphyes* | Volcano Darwin | VD | Isabela | 21 | 21 | 22 | 22 |
| *C. vanderburghi* | Volcano Alcedo | VA | Isabela | 28 | 24 | 28 | 24 |
| *C. guntheri* | La Cazuela | CAZ | Isabela | 22 | 23 | 22 | 23 |
| *C. vicina* | West Cerro Azul | LP | Isabela | 13 | 15 | 13 | 15 |
| *C. vicina* | West Cerro Azul | LT | Isabela | 12 | 13 | 12 | 13 |
| *C. becki* | Piedras Blancas | PBL | Isabela | 18 | 22 | 34 | 34 |
| *C. becki* | Puerto Bravo | PBR | Isabela | 27 | 29 | 29 | 42 |

**Table S5**: Pairwise F_ST_ values using mtDNA control region sequences (above the diagonal) or 12 microsatellite loci (below the diagonal). The two populations from *C. vicina* (LP/LT) were pooled together because they were found to be genetically part of the same population. Symbols and sample sizes are the same as in table S4. Values in bold represent comparisons among the three taxa considered in the main text.

|  | ESP | SCR | CF | CRU | PZ | AGO | VD | VA | CAZ | LP/LT | PBL | PBR |
| --- | --- | --- | --- | --- | --- | --- | --- | --- | --- | --- | --- | --- |
| ESP |  | 1.000 | 0.943 | 0.538 | 0.488 | 0.542 | 0.720 | 0.794 | 0.786 | 0.537 | 0.886 | 1.000 |
| SCR | 0.331 |  | 0.949 | 0.566 | 0.515 | 0.572 | 0.742 | 0.809 | 0.804 | 0.564 | 0.898 | 1.000 |
| CF | 0.426 | 0.292 |  | 0.526 | 0.478 | 0.530 | 0.699 | 0.770 | 0.761 | 0.525 | 0.848 | 0.957 |
| CRU | 0.290 | 0.162 | 0.164 |  | 0.150 | 0.177 | 0.340 | **0.439** | 0.405 | 0.189 | **0.466** | 0.613 |
| PZ | 0.342 | 0.243 | 0.342 | 0.218 |  | 0.145 | 0.303 | 0.398 | 0.365 | 0.158 | 0.422 | 0.560 |
| AGO | 0.286 | 0.158 | 0.250 | 0.119 | 0.177 |  | 0.338 | 0.440 | 0.404 | 0.184 | 0.468 | 0.621 |
| VD | 0.334 | 0.244 | 0.346 | 0.205 | 0.235 | 0.188 |  | 0.596 | 0.569 | 0.345 | 0.640 | 0.778 |
| VA | 0.329 | 0.234 | 0.318 | **0.202** | 0.223 | 0.186 | 0.105 |  | 0.654 | 0.441 | **0.721** | 0.833 |
| CAZ | 0.380 | 0.273 | 0.378 | 0.238 | 0.263 | 0.237 | 0.098 | 0.112 |  | 0.408 | 0.705 | 0.833 |
| LP/LT | 0.318 | 0.218 | 0.297 | 0.160 | 0.227 | 0.173 | 0.156 | 0.170 | 0.176 |  | 0.468 | 0.609 |
| PBL | 0.275 | 0.186 | 0.254 | **0.137** | 0.199 | 0.101 | 0.163 | **0.163** | 0.205 | 0.156 |  | 0.916 |
| PBR | 0.327 | 0.223 | 0.289 | 0.195 | 0.207 | 0.116 | 0.235 | 0.208 | 0.288 | 0.205 | 0.165 |  |

**Table S6**: Pairwise F_ST_ values after VA and CRU are combined into one “pseudo-population” using mtDNA control region sequences (above the diagonal) or microsatellite loci (below the diagonal). The two populations from *C. vicina* (LP/LT) were pooled together because they were found to be genetically part of the same population. Symbols and sample sizes are the same as in table S4. Values in bold represent comparisons among the three taxa considered in the main text.

|  | ESP | SCR | CF | VA+CRU | PZ | AGO | VD | CAZ | LP/LT | PBL | PBR |
| --- | --- | --- | --- | --- | --- | --- | --- | --- | --- | --- | --- |
| ESP |  | 1.000 | 0.943 | 0.501 | 0.488 | 0.542 | 0.720 | 0.786 | 0.537 | 0.886 | 1.000 |
| SCR | 0.331 |  | 0.949 | 0.518 | 0.515 | 0.572 | 0.742 | 0.804 | 0.564 | 0.898 | 1.000 |
| CF | 0.426 | 0.292 |  | 0.489 | 0.478 | 0.530 | 0.699 | 0.761 | 0.525 | 0.848 | 0.957 |
| VA+CRU | 0.239 | 0.141 | 0.177 |  | 0.182 | 0.207 | 0.347 | 0.398 | 0.218 | **0.446** | 0.548 |
| PZ | 0.342 | 0.243 | 0.342 | 0.164 |  | 0.145 | 0.303 | 0.365 | 0.158 | 0.422 | 0.560 |
| AGO | 0.286 | 0.158 | 0.250 | 0.100 | 0.177 |  | 0.338 | 0.404 | 0.184 | 0.468 | 0.621 |
| VD | 0.334 | 0.244 | 0.346 | 0.101 | 0.235 | 0.188 |  | 0.569 | 0.345 | 0.640 | 0.778 |
| CAZ | 0.380 | 0.273 | 0.378 | 0.121 | 0.263 | 0.237 | 0.098 |  | 0.408 | 0.705 | 0.833 |
| LP/LT | 0.318 | 0.218 | 0.297 | 0.108 | 0.227 | 0.173 | 0.156 | 0.176 |  | 0.468 | 0.609 |
| PBL | 0.275 | 0.186 | 0.254 | **0.094** | 0.199 | 0.101 | 0.163 | 0.205 | 0.156 |  | 0.916 |
| PBR | 0.327 | 0.223 | 0.289 | 0.146 | 0.207 | 0.116 | 0.235 | 0.288 | 0.205 | 0.165 |  |

**Table S7**: Pairwise F_ST_ values for mtDNA control region sequences (above the diagonal) or 12 microsatellite loci (below the diagonal). The two populations from *C. vicina* (LP/LT) were pooled together because they were found to be genetically part of the same population. This analysis also includes the 38 samples inferred to have admixed genotypes (see supplementary material text). Symbols and sample sizes are the same as in table S4.

|  | ESP | SCR | CF | CRU | PZ | AGO | VD | VA | CAZ | LP/LT | PBL | PBR |
| --- | --- | --- | --- | --- | --- | --- | --- | --- | --- | --- | --- | --- |
| ESP |  | 1.000 | 0.943 | 0.538 | 0.488 | 0.517 | 0.690 | 0.794 | 0.786 | 0.537 | 0.568 | 0.648 |
| SCR | 0.331 |  | 0.949 | 0.566 | 0.515 | 0.546 | 0.714 | 0.809 | 0.804 | 0.564 | 0.590 | 0.670 |
| CF | 0.426 | 0.292 |  | 0.526 | 0.478 | 0.506 | 0.672 | 0.770 | 0.761 | 0.525 | 0.556 | 0.633 |
| CRU | 0.290 | 0.162 | 0.164 |  | 0.150 | 0.162 | 0.319 | 0.439 | 0.405 | 0.189 | 0.248 | 0.309 |
| PZ | 0.342 | 0.243 | 0.342 | 0.218 |  | 0.131 | 0.283 | 0.398 | 0.365 | 0.158 | 0.216 | 0.275 |
| AGO | 0.286 | 0.163 | 0.251 | 0.119 | 0.176 |  | 0.299 | 0.420 | 0.385 | 0.170 | 0.229 | 0.290 |
| VD | 0.323 | 0.230 | 0.331 | 0.193 | 0.225 | 0.175 |  | 0.574 | 0.545 | 0.324 | 0.376 | 0.441 |
| VA | 0.329 | 0.234 | 0.318 | 0.202 | 0.223 | 0.184 | 0.101 |  | 0.654 | 0.441 | 0.480 | 0.548 |
| CAZ | 0.380 | 0.273 | 0.378 | 0.238 | 0.263 | 0.235 | 0.094 | 0.112 |  | 0.408 | 0.452 | 0.521 |
| LP/LT | 0.318 | 0.218 | 0.297 | 0.160 | 0.227 | 0.175 | 0.148 | 0.170 | 0.176 |  | 0.254 | 0.315 |
| PBL | 0.243 | 0.159 | 0.222 | 0.113 | 0.167 | 0.078 | 0.124 | 0.133 | 0.175 | 0.131 |  | 0.364 |
| PBR | 0.251 | 0.175 | 0.241 | 0.146 | 0.169 | 0.087 | 0.181 | 0.168 | 0.240 | 0.162 | 0.090 |  |

**Table S8**: Pairwise F_ST_ values after VA and CRU are combined into one “pseudo-population” using mtDNA control region sequences (above the diagonal) or microsatellite loci (below the diagonal). The two populations from *C. vicina* (LP/LT) were pooled together because they were found to be genetically part of the same population. This analysis also includes the 38 samples inferred to have admixed genotypes (see supplementary material text). Symbols and sample sizes are the same as in table S4.

|  | ESP | SCR | CF | VA+CRU | PZ | AGO | VD | CAZ | LP/LT | PBL | PBR |
| --- | --- | --- | --- | --- | --- | --- | --- | --- | --- | --- | --- |
| ESP |  | 1.000 | 0.943 | 0.501 | 0.488 | 0.517 | 0.690 | 0.786 | 0.537 | 0.568 | 0.648 |
| SCR | 0.331 |  | 0.949 | 0.518 | 0.515 | 0.546 | 0.714 | 0.804 | 0.564 | 0.590 | 0.670 |
| CF | 0.426 | 0.292 |  | 0.489 | 0.478 | 0.506 | 0.672 | 0.761 | 0.525 | 0.556 | 0.633 |
| VA+CRU | 0.239 | 0.141 | 0.177 |  | 0.182 | 0.193 | 0.330 | 0.398 | 0.218 | 0.269 | 0.321 |
| PZ | 0.342 | 0.243 | 0.342 | 0.164 |  | 0.131 | 0.283 | 0.365 | 0.158 | 0.216 | 0.275 |
| AGO | 0.286 | 0.163 | 0.251 | 0.098 | 0.176 |  | 0.299 | 0.385 | 0.170 | 0.229 | 0.290 |
| VD | 0.323 | 0.230 | 0.331 | 0.093 | 0.225 | 0.175 |  | 0.545 | 0.324 | 0.376 | 0.441 |
| CAZ | 0.380 | 0.273 | 0.378 | 0.121 | 0.263 | 0.235 | 0.094 |  | 0.408 | 0.452 | 0.521 |
| LP/LT | 0.318 | 0.218 | 0.297 | 0.108 | 0.227 | 0.175 | 0.148 | 0.176 |  | 0.254 | 0.315 |
| PBL | 0.243 | 0.159 | 0.222 | 0.071 | 0.167 | 0.078 | 0.124 | 0.175 | 0.131 |  | 0.364 |
| PBR | 0.251 | 0.175 | 0.241 | 0.106 | 0.169 | 0.087 | 0.181 | 0.240 | 0.162 | 0.090 |  |

**Figure S7**: **A.** A simplified phylogenetic tree of all extant and extinct species of Galápagos giant tortoises based on 124 mitochondrial DNA (mtDNA) control region haplotypes (redrawn from (Poulakakis et al. 2015)). The taxa considered in the main text are highlighted in bold: CRU = *C. porteri* from Santa Cruz island (La Caseta). VA = *C. vandenburghi* from central Isabela island (Volcano Alcedo), and PBL = *C. becki* from northern Isabela island (Volcano Wolf). Each triangle represents the clades including all the haplotypes from a given species, population location and island. The size of the triangles corresponds to the number of haplotypes. The colors in each triangle are the same as the genetic clusters identified by the Bayesian clustering analyses of microsatellite loci in panel B. Multiple colors in a triangle indicates that the clade includes haplotypes found in multiple STRUCTURE defined clusters, though note that colors are not proportional to their frequency in either analysis. The red scale bar near the root of the tree indicates expected changes per site. **B.** STRUCTURE bar plot indicating the genetic assignments of populations of all the extant and extinct species of Galápagos giant tortoises based on 12 microsatellite loci and 126 samples (redrawn from Miller et al., under review). Each individual is represented as a vertical bar, with colors denoting the different genetic clusters. The proportion of color in a bar is equal to the ancestry to a given cluster. The two or three letter symbols in each cluster refer to the populations for each species (Table S2), with the addition of PNT: *C. abingdoni*, Pinta Island, and FLO: *C. elephantopus*, Floreana Island. The populations symbols for the three species analyzed in this study are in bold.


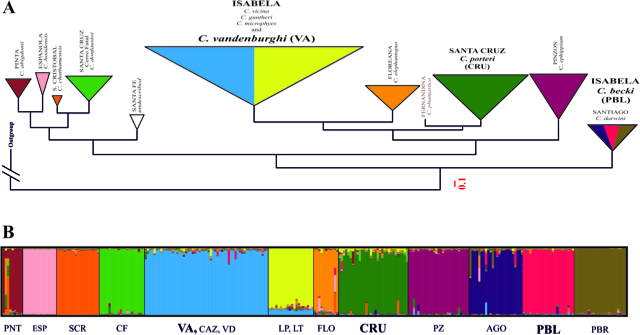


**Supplementary References**

Beheregaray LB, Ciofi C, Caccone A, Gibbs JP, Powell JR. 2003. Genetic divergence, phylogeography and conservation units of giant tortoises from Santa Cruz and Pinzon, Galapagos Islands. Conserv. Genet. 4:31–46.

Beheregaray LB, Ciofi C, Geist D, Gibbs JP, Caccone A, Powell JR. 2003. Genes record a prehistoric volcano eruption in the Galapagos. Science (80-. ). 302:75.

Beheregaray LB, Gibbs JP, Havill N, Fritts TH, Powell JR, Caccone A. 2004. Giant tortoises are not so slow: Rapid diversification and biogeographic consensus in the Galapagos. Proc. Natl. Acad. Sci. U. S. A. 101:6514–6519.

Benavides E, Russello M, Boyer D, Wiese RJ, Kajdacsi B, Marquez L, Garrick R, Caccone A. 2012. Lineage Identification and Genealogical Relationships Among Captive Galapagos Tortoises. Zoo Biol. 31:107–120.

Burns CE, Ciofi C, Beheregaray LB, Fritts TH, Gibbs JP, Marquez C, Milinkovitch MC, Powell JR, Caccone A. 2003. The origin of captive Galapagos tortoises based on DNA analysis: implications for the management of natural populations. Anim. Conserv. 6:329–337.

Caccone A, Gentile G, Gibbs JP, Fritts TH, Snell HL, Betts J, Powell JR. 2002. Phylogeography and history of giant Galapagos tortoises. Evolution (N. Y). 56:2052–2066.

Ciofi C, Milinkovitch MC, Gibbs JP, Caccone A, Powell JR. 2002. Microsatellite analysis of genetic divergence among populations of giant Galapagos tortoises. Mol. Ecol. 11:2265–2283.

Ciofi C, Wilson GA, Beheregaray LB, Marquez C, Gibbs JP, Tapia W, Snell HL, Caccone A, Powell JR. 2006. Phylogeographic history and gene flow among giant Galapagos tortoises on southern Isabela Island. Genetics 172:1727–1744.

Danecek P, Auton A, Abecasis G, Albers CA, Banks E, DePristo MA, Handsaker RE, Lunter G, Marth GT, Sherry ST, et al. 2011. The variant call format and VCFtools. Bioinformatics 27:2156–2158.

Edwards DL, Benavides E, Garrick RC, Gibbs JP, Russello MA, Dion KB, Hyseni C, Flanagan JP, Tapia W, Caccone A. 2013. The genetic legacy of Lonesome George survives: Giant tortoises with Pinta Island ancestry identified in Galapagos. Biol. Conserv. 157:225–228.

Edwards DL, Garrick RC, Tapia W, Caccone A. 2014. Cryptic structure and niche divergence within threatened Galápagos giant tortoises from southern Isabela Island. Conserv. Genet. 15:1357–1369.

Excoffier L, Laval G, Schneider S. 2005. Arlequin (version 3.0): An integrated software package for population genetics data analysis. Evol. Bioinforma. 1:47–50.

Garrick RC, Benavides E, Russello MA, Gibbs JR, Poulakakis N, Dion KB, Hyseni C, Kajdacsi B, Marquez L, Bahan S, et al. 2012. Genetic rediscovery of an “extinct” Galapagos giant tortoise species. Curr. Biol. 22:R10–R11.

Garrick RC, Benavides E, Russello MA, Hyseni C, Edwards DL, Gibbs JP, Tapia W, Ciofi C, Caccone A. 2014. Lineage fusion in Galapagos giant tortoises. Mol. Ecol. 23:5276–5290.

Garrick RC, Kajdacsi B, Russello MA, Benavides E, Hyseni C, Gibbs JP, Tapia W, Caccone A. 2015. Naturally rare versus newly rare: demographic inferences on two timescales inform conservation of Galápagos giant tortoises. Ecol. Evol. 5:676–694.

Goudet J. 2005. hierfstat, a package for r to compute and test hierarchical F-statistics. Mol. Ecol. Notes 5:184–186.

Kim D, Pertea G, Trapnell C, Pimentel H, Kelley R, Salzberg SL. 2013. TopHat2: accurate alignment of transcriptomes in the presence of insertions, deletions and gene fusions. Genome Biol. 14:R36.

Li H. 2011. A statistical framework for SNP calling, mutation discovery, association mapping and population genetical parameter estimation from sequencing data. Bioinformatics 27:2987–2993.

Loire E, Chiari Y, Bernard A, Cahais V, Romiguier J, Nabholz B, Lourenco JM, Galtier N. 2013. Population genomics of the endangered giant Galapagos tortoise. Genome Biol. 14.

Milinkovitch M, Monteyne D, Russello M, Gibbs J, Snell H, Tapia W, Marquez C, Caccone A, Powell J. 2007. Giant Galapagos tortoises; molecular genetic analyses identify a trans-island hybrid in a repatriation program of an endangered taxon. BMC Ecol. 7:doi:10.1186/1472-6785-7-2.

Milinkovitch MC, Monteyne D, Gibbs JP, Fritts TH, Tapia W, Snell HL, Tiedemann R, Caccone A, Powell JR. 2004. Genetic analysis of a successful repatriation programme: giant Galapagos tortoises. Proc. R. Soc. B-Biological Sci. 271:341–345.

Miller JM, Quinzin MC, Poulakakis N, Gibbs JP, Beheregaray LB, Garrick RC, Russello MA, Ciofi C, Edwards DL, Hunter EA, et al. Reviving a Lost Species: The Case of the Floreana Galápagos Giant Tortoise Chelonoidis elephantopus. Submitted.

Parent CE, Caccone A, Petren K. 2008. Colonization and diversification of Galapagos terrestrial fauna: a phylogenetic and biogeographical synthesis. Philos. Trans. R. Soc. B-Biological Sci. 363:3347–3361.

Poulakakis N, Edwards DL, Chiari Y, Garrick RC, Russello MA, Benavides E, Watkins-Colwell GJ, Glaberman S, Tapia W, Gibbs JP, et al. 2015. Description of a New Galapagos Giant Tortoise Species (Chelonoidis; Testudines: Testudinidae) from Cerro Fatal on Santa Cruz Island. PLoS One 10:e0138779.

Poulakakis N, Glaberman S, Russello M, Beheregaray LB, Ciofi C, Powell JR, Caccone A. 2008. Historical DNA analysis reveals living descendants of an extinct species of Galapagos tortoise. Proc. Natl. Acad. Sci. U. S. A. 105:15464–15469.

Poulakakis N, Russello M, Geist D, Caccone A. 2012. Unravelling the peculiarities of island life: vicariance, dispersal and the diversification of the extinct and extant giant Galapagos tortoises. Mol. Ecol. 21:160–173.

Raymond M, Rousset F. 1995. GENEPOP (version 1.2): Population genetics software for exact tests and ecumenicism. J Hered 86:248–249.

Rousset F. 2008. genepop’007: a complete re-implementation of the genepop software for Windows and Linux. Mol. Ecol. Resour. 8:103–106.

Russello MA, Beheregaray LB, Gibbs JP, Fritts T, Havill N, Powell JR, Caccone A. 2007. Lonesome George is not alone among Galápagos tortoises. Curr. Biol. 17:R317–R318.

Russello MA, Hyseni C, Gibbs JP, Cruz S, Marquez C, Tapia W, Velensky P, Powell JR, Caccone A. 2007. Lineage identification of Galapagos tortoises in captivity worldwide. Anim. Conserv. 10:304–311.
